# 

**Published:** 1840-04

**Authors:** 


### CONTENTS OF No. XVIII.

OF THE

### British and Foreign Medical Review.

APRIL, 1840.

### Part First.—Analytical and Critical Reviews.

PAGE

Art. I.—Recherches Cliniques sur l'Auscultation des Organes Respiratoires, et sur la Première Période de la Phthisie Pulmonaire. Par Jules Fournet . 299

Clinical Researches on Auscultation of the Respiratory Organs, and on the First Stage of Pulmonary Phthisis. By Jules Fournet.

Art. II.—First Annual Report of the Registrar-General of Births, Deaths, and Marriages in England . . . . . . 344

Art. III.—De Chemicis Calculorum Vesicariorum Rationibus. Scripsit E. A. Scharling, a.a., l.l., M. Chemiæ Lector . . . . 360
On the Chemical Relations of Urinary Calculi. By E. A. Scharling, Lecturer on Chemistry, &c.

Art. IV.—Guy's Hospital Reports. Nos. VIII.-IX. April and October, 1839 . . . . . . . 370

Art. V.—Pathologische Untersuchungen. Von Dr. Henle, Prosector und Privatdocenten in Berlin. . . . . . . 398

Pathological Investigations. By Dr. Henle.

Art. VI.—Traité des Etudes Médicales, ou de la Manière d'étudier et d'enseigner la Médecine. Par E. Fred. Dubois (d'Amiens), Professeur agrégé à la Faculté de Médecine de Paris, &c. &c. . . . . 411

A Treatise on Medical Study, or on the Method of Studying and of Teaching Medicine. By E. Fred. Dubois (of Amiens.)

Art. VII.—Medico-Chirurgical Transactions, published by the Royal Medical and Chirurgical Society of London. Second Series. Vol. IV. . . 431

Art. VIII.—1. On Inflammation, Chronic Disease, and Perforative Ulceration of the Cæcum, and of the Appendix Vermiformis Cæci, with Symptomatic Peritonitis and Fæcal Abscess. By John Burne, m.d. (Med. Chir. Trans., Vol. II.) . . . . . . 445
2. Memoir on Tuphlo-Enteritis; or Inflammation and Perforative Ulceration of the Cæcum, and of the Appendix Vermiformis Cæci. By John Burne, m.d. (Med. Chir. Trans., Vol. IV.) . . . . . . ib.
3. Beobachtungen auf dem Gebiete der Pathologie und Pathologischen Anatomie. Von Dr. J. F. Albers. . . . . . . ib.
Observations in Pathology and Pathological Anatomy. (Chapter on Typhlitis.) By Dr. J. F. Albers.
4. Histoire de Tumeurs Phlegmoneuses de Fosses Iliaques. Par le Docteur Grisolle. (Archives Générales de Médecine.) . . . ib.
History of Phlegmonous Tumours of the Iliac Fossæ. By Dr. Grisolle.

Art. IX.—1. The Principles and Practice of Medicine; founded on the most extensive experience in public hospitals and private practice; and developed in a course of Lectures delivered at University College, London. By John Elliotson, m.d., f.r.s., &c. With Notes and Illustrations, by Nathaniel Rogers, m.d. . . . . . . . 461
2. Lectures on the Theory and Practice of Medicine, delivered in University College, London. By John Elliotson, m.d., f.r.s., &c. Edited by J. C. Cooke, m.d., and T. C. Thompson, m.d. . . . . ib.
3. Clinical Lectures, delivered during the Sessions 1834-5 and 1836-7. By Robert J. Graves, m.d., &c. (American Medical Library.) . . ib.
4. Lectures on the Theory and Practice of Medicine. By William Stokes, m.d., &c. (American Medical Library.) . . . . ib.

PAGE

5. Elements of the Practice of Medicine. By RICHARD BRIGHT, M.D., and THOMAS ADDISON, M.D. . . . . . . 461
6. Elements of the Practice of Medicine, designed as a Text-Book for the use of Students. By WILLIAM REID, M.D. . . . . . ib.
7. Elements of the Theory and Practice of Medicine, designed for the use of Students and Junior Practitioners. By GEORGE GREGORY, M.D., &c. . ib.

ART. X.—Mikroskopische Untersuchungen über die Uebereinstimmung in der Struktur und dem Wachsthum der Thiere und Pflanzen. Von Dr. TH. SCHWANN . . . . . . . . 495
Microscopical Researches respecting the Identity of Structure and mode of Growth in Animals and Plants. By Dr. THOMAS SCHWANN. (With two Plates.)

---

### PART SECOND.—Bibliographical Notices.

ART. I.—Elements of Natural Philosophy; being an Experimental Introduction to the Study of the Physical Sciences. By GOLDING BIRD, M.D., F.L.S., F.G.S., &c. . . . . . . . 529

ART. II.—Observations on Yaws, and its Influence in Originating Leprosy; also Observations on Acute Traumatic Tetanus, and Tetanus Infantum. By JAMES MAXWELL, M.D. . . . . . . . 531

ART. III.—The Intimate Structure of the Secreting Glands, by JOHN MÜLLER, M.D., with the subsequent discoveries of other Authors. By SAMUEL SOLLY, F.R.S., Lecturer on Physiology, and on Comparative Anatomy, at St. Thomas's Hospital, &c. . . . . . . 533

ART. IV.—Insecta. By GEORGE NEWPORT, Esq. (Cyclopædia of Anatomy and Physiology) . . . . . . . ib.

ART. V.—Outlines of Physiology; with an Appendix on Phrenology. By P. M. ROGET, M.D., Secretary to the Royal Society, &c. . . . 534

ART. VI.—Rudiments of Animal Physiology, for Use in Schools, and for Private Instruction. By Dr. G. HAMILTON, Falkirk. (In Chambers's Educational Course.) . . . . . . . 535

ART. VII.—Animal Mechanism and Physiology. By JOHN H. GRISCOM, M.D., Professor of Chemistry in the New York College of Pharmacy . . 537

ART. VIII.—On the Anatomy of the Breast. By Sir ASTLEY P. COOPER, Bart., F.R.S., D.C.L., G.C.H., Serjeant-Surgeon to the Queen, &c. &c. . . 538

ART. VIII.*—1. Anatomical Tables; containing Concise Descriptions of the Muscles, Ligaments, Fasciæ, Blood-vessels, and Nerves. Intended for the Use of Students. By THOMAS NUNNELEY, Lecturer on Anatomy and Physiology in the Leeds School of Medicine, &c. . . . . ib.
2. Text-Book of Anatomy for Junior Students. By ALEX. J. LIZARS, M.D., Lecturer on Anatomy in Edinburgh, &c. . . . . . ib.
3. The Anatomist's Manual; or, a Treatise on the Manner of Preparing all the Parts of Anatomy, followed by a complete description of all these parts. By J. P. MAYGRIER, M.D.P., &c. . . . . . . ib.

ART. IX.—The Cyclopædia of Practical Surgery. Edited by W. B. COSTELLO, M.D. Parts IV-V. . . . . . . . 539

ART. X.—1. An Atlas of Plates Illustrative of the Principles and Practice of Obstetric Medicine and Surgery. With Descriptive Letterpress. By F. H. RAMSBOTHAM, M.D., Physician to the Royal Maternity Charity, &c. Nos. I., II., and III. . . . . . . . 540
2. Illustrations of Midwifery; a Complete Atlas and Companion to all Obstetric Works. By M. RYAN, M.D., &c. Parts I.-V. . . . ib.

ART. XI.—1. New Remedies: the method of preparing and administering them; their effects on the diseased and healthy economy, &c. By ROBLEY DUNGLISON, M.D., Professor of the Institutes of Medicine in Philadelphia College . . . . . . . . 541

PAGE

2. A Compendium of Materia Medica and Pharmacy, adapted to the London Pharmacopœia, embodying all the new French, American, and Indian Medicines, &c. By J. H. Lane, M.D., &c. . . . . 541
3. A Compendium of Materia Medica, Pharmacy, and Toxicology. For the Use of Medical Students. By D. Spillan, M.D. . . ib.

Art. XII.—Proceedings of the Border Medical Society. Instituted June 27, 1838. Parts I. and II. . . . . . . 542

Art. XIII.—A Practical Treatise on the Diseases of the Eye. By William Mackenzie, M.D. To which is prefixed an Anatomical Introduction, Explanatory of a Horizontal Section of the Human Eyeball. By Thomas Wharton Jones, Surgeon . . . . . 543

Art. XIV.—The Medical Portrait Gallery. By T. J. Pettigrew, F.R.S. Vol. IV. ib.

Art. XV.—Observations on the Management of the Poor in Scotland, and its Effects on the Health of the Great Towns. By W. P. Alison, M.D. . 544

### Part Third.—Selections from the British and Foreign Journals.

### I. THE FOREIGN JOURNALS.

### ANATOMY AND PHYSIOLOGY.

Dr. Remak on the Injurious Qualities of Menstrual Blood, and on its Probable Causes . . . . . . . 545
MM. Vrolik and Vriese on the Evolution of Heat in Plants . . . ib.
Dr. Kronenberg's Experiments on the Motor and Sensitive Roots of the Nerves . 547
Dr. Pappenheim on the Nerves of the Cornea . . . . 548

### PATHOLOGY, PRACTICAL MEDICINE, AND THERAPEUTICS.

M. Dubois on Aneurism of the Ascending Aorta . . . . ib.
M. Vernois on the Various Circumstances which appear, in the Course of Diseases, to produce the Curved Form of the Nails . . . . ib.
Dr. Holscher on Apoplexy of the Eyes . . . . . 549
Prof. Bouillaud's New Clinical Researches on Sanguineous Concretions formed during life in the Heart and large Vessels . . . . ib.
Dr. Hannover on a Contagious Growth of Conferva on the Water Salamander (Triton punctatis) . . . . . . 550
Dr. Kreysig on a Remarkable Case of Ischuria Renalis, of Nine Years' Standing, with Vicarious Vomiting of Urine . . . . . 551
Dr. Payan on the Use of Hydrochlorate of Baryta in Strumous Ophthalmia . 552
M. Saucerotte on the Treatment of Dysentery with Albumen . . ib.
M. Taufflied on the Employment of the Oil of Cod Fish in Scrofulous Diseases . 553

### SURGERY.

M. Ordinaire on the Treatment of Malignant Ulcers by Cauterization with Corrosive Sublimate . . . . . . . ib.
M. Barthelemy on the Treatment of Synovial Tumours by Subcutaneous Incision 554
M. Bouvier on Division of the Muscles of the Back in Cases of Lateral Deviation of the Spine . . . . . . . . ib.
Prof. Dieffenbach on the Cure of an Old Dislocation of the Humerus by Division of the Pectoralis Major, Latissimus Dorsi, Teres Major, and Teres Minor Muscles . . . . . . . 555

PAGE

M. Richet's case of Dislocation of the Elbow, in which the Radius and Ulna were thrown forwards, with Fracture of the Ulna . . . . 556
M. Caffe's Memoir on the Ophthalmia reigning in Belgium . . . 557
Prof. Dieffenbach on the Cure of Congenital Squinting by Division of the Internal Straight Muscle of the Eye . . . . . 558
Dr. E. Laborie's Cases in Autoplastic Surgery—Formation of New Eyelid . 559

MIDWIFERY.

M. Piedagnel on the Influence of Digitalis in the Contractions of the Uterus . 560

II. THE AMERICAN AND COLONIAL JOURNALS.

ANATOMY AND PHYSIOLOGY.

Drs. Pennock and Moore's Report of Experiments on the Action of the Heart . 561
Dr. Cottman on the Sedative Effects of Ergot (Experiments with the Powder) . 562

PATHOLOGY, PRACTICAL MEDICINE, AND THERAPEUTICS.

Dr. Buchanan's case illustrative of the Etiology of Spontaneous Amputation of the Limbs of the Fœtus in Utero . . . . . 564
Dr. Mouat on the Causes of the Acute Diseases of European Soldiers in India . ib.

III. THE BRITISH JOURNALS.

ANATOMY AND PHYSIOLOGY.

Dr. Todd on the Structure of the Mucous Membrane of the Stomach . . 565
Mr. Blake's Observations and Experiments on the Mode in which various Poisonous Agents act on the Animal Body . . . . . 569
Dr. Paterson's Observations on Corpora Lutea . . . . 570
Dr. Reid's four cases of Aneurism of the Arch of the Aorta, and a case of Diaphragmatic Hernia . . . . . 571

PATHOLOGY, PRACTICAL MEDICINE, AND THERAPEUTICS.

Dr. Paley's cases of Mortification of the Male Genitals, giving rise (as is supposed) to fatal Fever by Infection . . . . . ib.
Mr. Campbell's Cases and Observations on Anuria Apyretica . . . 573
Dr. Wilson on the Treatment of Croup by large doses of Tartar Emetic . . ib.
Mr. Green's contributions to the Pathology of Children . . . 574

SURGERY.

Mr. Dodd on the Treatment of Varicose Veins . . . . 575

Dr. Osborne on Thread Setons . . . . . . 578

Mr. Kennedy's Observations on Diffuse Inflammation . . . ib.

PART FOURTH.—Medical Intelligence.

On the Torula Cerevisiæ, or Yeast-Plant . . . . . 57[illegible]
Dr. Hall's Enquiries into the Nervous System . . . . 580
New Bills of Mortality for London . . . . . 582
Books received for Review . . . . . . 583

Index, Title, &c.

